# Could Polyphenols Help in the Control of Rheumatoid Arthritis?

**DOI:** 10.3390/molecules24081589

**Published:** 2019-04-22

**Authors:** Siyun Sung, Doyoung Kwon, Eunsik Um, Bonglee Kim

**Affiliations:** 1 College of Korean Medicine, Kyung Hee University, Seoul 02453, Korea; stellasung95@khu.ac.kr (S.S.); doyoung@khu.ac.kr (D.K.); 2 Department of Clinical Korean Medicine, Graduate School, Kyung Hee University, Seoul 02453, Korea; flare0722@khu.ac.kr; 3 Department of Pathology, College of Korean Medicine, Graduate School, Kyung Hee University, Seoul 02453, Korea

**Keywords:** rheumatoid arthritis, natural products, polyphenol, flavonoids, phenolic acid, stilbene

## Abstract

Rheumatoid arthritis (RA) is a chronic, systemic, joint-invading, autoimmune inflammatory disease, which causes joint cartilage breakdown and bone damage, resulting in functional impairment and deformation of the joints. The percentage of RA patients has been rising and RA represents a substantial burden for patients around the world. Despite the development of many RA therapies, because of the side effects and low effectiveness of conventional drugs, patients still need and researchers are seeking new therapeutic alternatives. Polyphenols extracted from natural products are effective on several inflammatory diseases, including RA. In this review polyphenols are classified into four types: flavonoids, phenolic acids, stilbenes and others, among which mainly flavonoids are discussed. Researchers have reported that anti-RA efficacies of polyphenols are based mainly on three mechanisms: their anti-inflammatory, antioxidant and apoptotic properties. The main RA factors modified by polyphenols are mitogen-activated protein kinase (MAPK), interleukin-1β (IL-1β), IL-6, tumor necrosis factor-α (TNF-α), nuclear factor κ light chain enhancer of activated B cells (NF-κB) and c-Jun N-terminal kinases (JNK). Polyphenols could be potent alternative RA therapies and sources for novel drugs for RA by affecting its key mechanisms.

## 1. Introduction

Rheumatoid arthritis (RA) is a notorious chronic autoimmune inflammatory joint disease, which can cause cartilage and bone damage [1]. This disease is characterized by synovial inflammation, swelling, autoantibody production, cartilage and bone destruction, and systemic features such as cardiovascular, pulmonary, and skeletal disorders. It is associated with progressive disability, systemic complications, early death and socioeconomic costs [2]. As of 2015 is estimated that RA affects about 24.5 million people [3]. This number includes 0.5 to 1% of adults in the developed world, 5 to 50 per 100,000 patients newly added each year [1]. Although the critical damage caused by this disease is well known and thus widely studied, the mechanism(s), underlying cause and pathway(s) of RA are not well-known. 

The number of therapeutic solutions available for treating RA has continuously grown in the past 30 years. These solutions include non-steroidal anti-inflammatory drugs, glucocorticoids, disease-modifying anti-rheumatic drugs (DMARDs) of synthetic origin (e.g., methotrexate and c-Jun N-terminal kinase (JNK) inhibitors) and of biological origin (ex. tumor necrosis factor (TNF) inhibitors, interleukin (IL)-6 inhibitor, and B cell-depleting drugs) [4]. Recently, medications that suppress the Janus kinase (JAK) pathways have shown noticeable effects as RA treatments, showing higher efficacy compared to the traditional ones. Tofacitinib and baricitinib, especially, are among the medications that show the most considerable effect and therefore have been extensively studied in clinical trial programs [4]. However, traditional DMARDs frequently present side-effects such as cytopenia, transaminase elevation, and poor tolerability. Another class of newly emerging solutions, the JAK inhibitors, also often cause gastrointestinal side-effects, lymphopenia, neutropenia, elevated cholesterol, and more infections [4]. On the basis of micro-environmental changes, severe synovial systematical reorganization and local fibroblast activation trigger synovial inflammation occur in RA [5]. The essential triggers of RA are unknown, but several genetic loci related to RA have been found [6]. These include major histocompatibility complex, class II, DR beta 1 (HLA-DRB1), Signal transducer and activator of transcription 4 (STAT4), protein tyrosine phosphatase (PTPN22), peptidyl arginine deiminase type I, IV (PAD14), and cytotoxic T-lymphocyte antigen 4 (CTLA4) [7]. Environmental factors such as smoking may stimulate the development of the disease by modifying genetic factors, but the specific mechanism(s) remain unknown [7]. 

Interactions of T cells, B cells, and related cytokines play key roles in developing RA symptoms such as synovitis, bone destruction, and cartilage degradation. The major cytokines that play a significant role in this process are TNFα, IL-6, IL-1, and IL-17 [8]. Like other auto-immune diseases, no perfect medications to treat RA have been developed. Recently many researchers have been trying to develop solutions for RA from natural products which have low toxicity and therefore assumed to have less side-effects. Polyphenols is one of the major classes of natural products that have been studied in this context. They are plant secondary metabolites that normally play a role in blocking ultraviolet radiation or pathogens. Numerous studies have shown that polyphenol-rich diets exert cardioprotective, anti-cancer, anti-diabetic and anti-aging effects [9]. Recognizing the strong anti-inflammatory effect of polyphenols and its potential role as a treatment for RA, we review herein the literature works that elucidate the effects of polyphenols on RA. 

## 2. Polyphenols and Rheumatoid Arthritis

### 2.1. Phenolic Acids

Hydroxybenzoic and hydroxycinnamic acids are characteristic phenolic acids. Phenolic acids account for about a third of the polyphenolic compounds in our diet and are found in all plant material, but they are particularly abundant in acidic-tasting fruits. Caffeic acid, gallic acid, and ferulic acid are some common phenolic acids. Phenolic acids showing anti-RA effects are arranged in Table 1. When monocyte and macrophage cells from rat were pre-exposed for 24 h to ferulic acid, which is found in grains, vegetables, fruits and nuts, nuclear factor of activated T cells c1 (NFATc1), c-Fos, NF-κB, tartrate-resistant acid phosphatase (TRAP), matrix metalloproteinases (MMP)-9, Cathepsin activities were depressed [10]. The natural polyphenol N-feruloylserotonin (N-f-5HT), extracted from *Leuzea carthamoides*, had RA-inhibitory effects via suppressing c-reactive protein (CRP), 12/15-lipoxygenase (LOX), TNF-α, inducible nitric oxide synthase (iNOS), IL-1β in liver and spleen cells of arthritic rats. This study was conducted for 28 days, with 3 mg/kg of N-f-5HT [11]. In the study of Lee, mRNA transcription of TNF-α was significantly attenuated in a human mast cell line (HMC-1) treated with gallotanin derived from *Euphorbia* [12]. Chlorogenic acid (CGA), derived from *Gardenia jasminoides*, inhibited the phosphorylation of p38, Akt, extracellular signal-regulated kinase (ERK) and IkB, also suppressed the mRNA expression of nuclear factor activated T cells cl (NFATcl). Furthermore, lipopolysaccharide (LPS)-induced bone erosion was alleviated in vivo when bone marrow macrophages (BMMs) were exposed to 10, 25, 50 μg/mM of CGA for 4 days [13]. *p*-Coumaric Acid (CA), which can be extracted from *Gnetm cleistostachyum*, was used in two studies. Both of them used the same dose of 100 mg/kg of CA to treat an adjuvant-induced arthritis (AIA) rat model. One trial with a duration of 8 days presented degradation of TNF-α and circulating immune complexes (CIC) levels while inducing alleviation of immunoglobulin G (IgG) [14]. In the other 16 day trial, CA treatment also reduced TNF-α activation, suggest an anti-RA effect via attenuation of cytokines, chemokines, osteoclastogenic factors, transcription factors, and mitogen-activated protein kinase (MAPK). In detail, the affected cytokines and chemokines are IL-1β, IL-6, monocyte chemoattractant protein (MCP)-1, the osteoclast factors are receptor activator of nuclear factor kappa-B ligand (RANKL), TRAP, the pro-inflammatory cytokines are IL-1b, IL-6, IL-17, the inflammatory enzymes are iNOS and cyclooxygenase (COX)-2, the transcription factors are NF-κB-p65, p-NF-κB-p65, NFATc-1, c-Fos, MAP kinases are JNK, p-JNK, ERK1/2. However, osteoprotegerin (OPG) elevation was shown [15].

### 2.2. Stilbenes

Stilbenes have a 1,2-diphenylethylene nucleus that can be of two types: (*E*)-stilbenes which are the *trans* isomers and (*Z*)-stilbenes which are *cis* isomers [17]. Stilbenes are polyphenols with anti-inflammatory, cell death activation, and anti-oxidant effects. Among more than 400 natural stilbenes, the most popular one is resveratrol (RSV). RSV was reported as a new potential agent to suppress inflammation-induced arthritis (Table 2). RSV, which is originated from red grapes, showed anti-RA effect on FLSs of AA that was given with a dose of 5, 15, 45 mg/kg of the compound for 12 days, by inhibiting Beclin1, LC3A/B, manganese-dependent superoxide dismutase (MnSOD) and inducing MtROS [18]. A dose on FLSs in humans of 50 μg for 24 h also demonstrated an anti-RA effect via suppression of COX-2, prostaglandin E2 (PGE2), nicotinamide adenine dinucleotide phosphate (NADPH) oxidase, Akt, p38 MAPK, ERK1/2, reactive oxygen species (ROS), NF-κB [19]. On human synovial membrane in a test conducted with resveratrol at a dose of 6.25, 12.5, 25, 50 µM, resveratrol exerted the same effect by regulating IL-1β, MMP-3, p-Akt, MMP-3, PI3K-Akt [20]. In the randomized controlled clinical trial by Hani, 50 patients were given a 1 g RSV capsule for 3 months. This study suggested that taking RSV has significant clinical effect in RA. Also, RF positivity, SJC-28, TJC-28, CRP, erythrocyte sedimentation rate (ESR), uncarboxylated osteocalcin (ucOC), MMP-3, TNF-α, IL-6, disease Activity Score-28 for Rheumatoid Arthritis with ESR (DAS28-ESR) levels were alleviated [21]. Furthermore, RSV relieved RA symptoms by downregulating IgG1, IgG2a when a dose of 20 mg/kg was used. After treatment of draining lymph node (DLN) cells and Th17 cells of rat with 40 µM of RSV for 72 h, expressions of IL-17 and IFN-γ were decreased. With the same cell line, injection of 30 µM or 50 µM for 3 days led to suppression of TH-17, IL-17 [22]. Finally, RSV-exposed FLSs in AA showed a decline of Beclin1, LC3A/B, MnSOD and increase of mitochondrial (Mt) ROS [23].

### 2.3. Flavonoids

Flavonoids are a type of polyphenol which consist of two phenyl rings in a general 15-carbon skeleton structure. They can be classified into flavones, flavonols, flavanones, flavanonols, flavanols or catechins, anthocyanins, and chalcones [24]. Quercetin and epigallocatechin-3-gallate, a tea flavonoid, are some of the best known flavonoids. These compounds have beneficial effects such as anti-inflammatory and anti-cholinesterase activity and therefore are used to treat many diseases. For example, a flavonoid-rich diet was reported to be associated with a reduced risk of cardiovascular disease [25]. Citrus flavonoids can modulate lipid metabolism and thus can be used as a treatment of metabolic dysregulation [26]. The anti-inflammatory effects of flavonoids can also be applied to attenuating the symptoms of rheumatoid arthritis (Table 3). A-glucosylhesperidin is extracted from citrus fruits, and exerts anti-RA effects via downregulation of tumor necrosis factor α (TNFα) at a dose of 3 mg per 0.3 mL when it was administered on a collagen-induced arthritis (CIA) rat model 3 times a week for 31 days [27]. Anthocyanin from cherries showed anti-RA effects by inhibiting TNFα, prostaglandin E2 (PGE2), and malondialdehyde (MDA) and inducing superoxide dismutase (SOD) at doses of 10, 20, and 40 mg/kg when adjuvant induced arthritis (AIA) rats were treated for 14 days [28]. Also, cocoa polyphenol, which consists of epicatechin, catechin, flavonol glycosides, and procyanidin, downregulated vascular endothelial growth factor (VEGF), NF-kB, and activator protein (AP)-1 and increased formation of p-Akt, p-p70S6K, p-extracellular signal-regulated kinases (ERK), p-p90 kDa ribosomal S6 kinase (p90RSK), p-mitogen-activated protein kinase kinase 4 (MKK4), p-c-Jun N-terminal kinase (JNK), p- PI3K when a JB6 P+ mouse epidermal cell model was treated with doses of 10 and 20 μM /mL for 1 h [29]. Epigallocatechin-3-gallate (EGCG), a well-known compound from Camellia sinensis, exerted anti-RA effects on human rheumatoid arthritis synovial fibroblasts (RASF) by downregulating epithelial neutrophil-activating peptide (ENA)-78, RANTES, growth-regulated oncogene (GRO)-α, IL-1–induced MMP-2, chemokine-induced MMP-2 at doses of 10, 20, 30, 40 and 50 μM when administered for 12 h [30]. Doses of 125, 250, 500 nM of EGCG for 24 h also demonstrated anti-RA effects on human rheumatoid arthritis synovial fibroblasts (RASFs) via suppression of mitogen-activated protein kinase (MAPK), MMP-1, MMP-3, p-extracellular regulated kinases (ERK)1/2, p-JNK, p-p38, and AP-1 formation [31]. When EGCG was given to CIA rats for 3 weeks, at a dose of 20, 30, 40, and 50 mg/kg, it inhibited type II collagen (CII) antigen-specific IgG2a, IL-1β, IL-6, TNFα, IL-17, VEGF, nitrotyrosine, iNOS, c-Fos, nuclear factor of activated T cells c1 (NFATc1), cathepsin K (CTSK), MMP9, p-STAT3 727, IL-17, chemokine (C-C motif) ligand 6 (CCL6), aryl hydrocarbon receptor (AHR), IL-21, p-STAT3 705, p-ERK, receptor activator of nuclear factor κ B (RANK), tartrate-resistant acid phosphatase (TRAP), and calcitonin receptor (CTR), while it induced IL-10, TGF-β, suppressor of cytokine signaling 3 (SOCS3), Foxp3 [32]. Furthermore, IL-6, TNFα, and interferon (IFN)-γ were suppressed, but anti-CII specific IgG1 antibodies were activated when CIA rats were treated for 3 weeks with a dose of 10 mg per kg of the rats’ weight [33]. EGCG at doses of 10 mg/kg for 5 days also showed anti-RA effects on pristane-induced arthritic (PIA) rats via inhibition of myeloperoxidase (MPO) [34]. When both human osteoclasts of peripheral blood monocytes and mice were treated for 15 days with a dose of 20 and 50 μM, CTR, carbonic anhydrase II, cathepsin K, α-v integrin, β-3 integrin, and NF-ATc1 were downregulated [35]. On osteoclast precursor cells and mature rat osteoclasts, 7 days of EGCG treatment with a dose of 10 and 100 μM restrained multinucleated osteoclast formation, MMP-9, and MMP-2, showing anti-RA effects [36]. Another flavonoid, fisetin from Rhus verniciflua Stokes, displayed anti-RA effects on human RA fibroblast-like synoviocytes (RAFLS) when they were treated with a dose of 0.1, 1, or 10 μg/mL for 72 h. FLS proliferation, TNFα, IL-6, IL-8, monocyte chemoattractant protein (MCP)-1, and VEGF were suppressed [37]. Under the same conditions described above, a flavonol-rich residual layer of the hexane fraction (RVHxR) derived from Rhus verniciflua Stokes, also inhibited FLS proliferation, TNFα, IL-6, IL-8, MCP-1, and VEGF and further inhibited p-ERK and p-JNK, while upregulated p-p38-MAPK [37]. Gallic acid, extracted from Cinnamomum zeylanicum L. bark, reduced RA symptoms by suppressing TNFα expression on adjuvant-induced arthritis (AIA) rats at a dose of 200 mg/kg for 12 and 21 days. When concanavalin (Con-A)-stimulated lymphocytes were treated at a dose of 40 μg/100 μL for 72 h, IL-2, IL-4, and IFN-γ were repressed [38]. One of the main flavonoids in soybean is genistein, which is reported to have anti-RA effects on CIA rat by reducing IFN-γ and T-bet, and the Th1/Th2 ratio, while it upregulates GATA-3 and IL-4. These effects were demonstrated in a dose of 1 mg/kg rat weight after treatment for 42 days [39]. Genistein also exerted the same effect via inhibition of FLS proliferation and MMP-9 when tested on human RAFLS for 24 h at a dose of 10 μg/mL [40]. Hesperidin ameliorated RA symptoms, inhibiting ELA, TBAR, and nitrite (NO), while inducing glutathione (GSH), SOD, and catalase when CIA rats were treated for 22 days with a dose of 160 mg/kg [41]. Kaempferol (3,4′,5,7-tetrahydroxyflavone), which is derived from diverse sources such as propolis and grapefruits, presented anti-RA effects on synovial tissues of patients with knee arthroplasty. When 10, 50, 100, and 200 μM of the flavonoid were applied on the tissue for 2 days, MAPK, NF-κB, and RASFs were inhibited. When a dose of 100 μM of the compound was applied on the tissue for 48 h, MAPK, NF-κB, MMP-1, MMP-3, COX-2, and PGE2 were inhibited [16]. Malvidin-3-O-β glucoside, extracted from red grape skin extract powder, relieved RA symptoms by downregulating TNF-α, IL1, macrophage inflammatory protein 1a (MIP1a), IL-8, IL-6, NO, and NOx when a dose of 1, 10, and 100 μM was applied to human peripheral blood monocyte-derived macrophages for 24 h. When peritoneal macrophages of rat were treated under the same conditions IL-1β, TNF-α, and IL-8 were suppressed [42]. Mangiferin (1,3,6,7-tetrahydroxyxanthone-C2-β-D-glucoside), derived from the family Thymelaeaceae, showed anti-RA effects via suppression of NF-κB, ERK1/2, IL-1β, IL-6, TNF-α, and RANKL when it was tested on CIA-induced DBA/1 rats at doses of 100 and 400 mg/kg for both 14 days and 27 days [43]. Morin (ML-morin) from various fruits and vegetables reduced RA symptoms by decreasing ROS, NO, iNOS, NF-κB-p65, TNF-α, IL-1-beta, IL-6, MCP-1, VEGF, RANKL, and STAT-3 formation in spleen and synovial macrophages of Wistar albino rats when they were treated with a dose of 10 mg/kg for 3 days [44]. Naringin, which can be extracted from grapes and citrus fruits, presented anti-RA effects on AIA rat by reducing TNFα, IL-1β, IL-6, and Bcl-2 formation while increasing Bax formation. The rats were treated with the compound at a dose of 20 mg/kg and of 40 mg/kg for 28 days, and both doses showed similar results [45]. Theaflavin-3,3′-digallate (TFDG), derived from Camellia sinensis exerted anti-RA effects via downregulation of multinucleated osteoclast formation, MMP-9 and MMP-2 of osteoclast precursor cells and mature osteoclast of rats treated with a dose of 10 and 100 μM for 7 days [36]. Thymoquinone (TQ) is extracted from Nigella sativa, and induces anti-RA effects by downregulating IL-6, IL-8, intercellular adhesion molecules (ICAM)-1, vascular cell adhesion protein (VCAM)-1, Cad-11, p38, and JNK in human RA synovium which was treated with 1, 2, 3, 4, and 5 μM of the compound for 2 h [46]. A similar effect was shown through a different mechanism when it was administered to CIA rats at 2.5 mg/kg for 5 days: TQ reduced IL-1β formation in CIA rat [47]. TQ also inhibited LPS-induced FLS proliferation, LPS-induced IL-1β, TNFα, MMP-13, COX-2, prostaglandin, H_2_O_2_-induced 4-hydroxynonenal (HNE), p-p38 -MKK, p-ERK, and p-NF-κB-p65 in human RAFLS treated with 1, 2, 5, and 10 μM of the compound for 1 h. When it was given to AIA rats at a dose of 5 mg/kg for 1 day, an anti-RA effect appeared via downregulation of HNE, IL-1β, and TNFα [48].

### 2.4. Other Compounds

Other polyphenols were also studied for their anti-RA mechanisms (Table 4). EVOO-polyphenol extract (PE), which is extracted from extra virgin oil (EVOO), exerts anti-RA effects via downregulation of TNF-α, IL-1β, IL-6, PEG2, p38, JNK, p65, and lκB-α at a dose of 100 and 200 mg/kg when collagen-induced arthritis (CIA) rats were treated for 13 days [49]. Hydroxytyrosol acetate (Hty-Ac), also from EVOO, showed anti-RA effects by inhibiting IgG1, IgG2a, COMP, MMP-3, TNF-Q, IFN-S, IL-1R, IL-6, IL-17A, nuclear factor (erythroid-derived 2)-like 2 (Nrf2), and heme oxygenase 1 (HO-1) at a dose of 0.5% when CIA rats were treated for 42 days [50]. Curcuminoid from turmeric rhizome or ginger rhizome, induced TNF-α, IL-1β, IL-6, IL-4, IL-10, SOD, CAT, and GSH, while suppressing lipid peroxidation (LPO), alanine transaminase (ALAT), and alkaline phosphatase (ALP) when it was given to AIA rats at a dose of 200 mg/kg/28 days [51]. Curcumin, also from the same origin, relieved RA symptoms of MH7A via downregulation of IL-1β, IL-6, NF-κB, ERK1/2, and AP-1, and upregulation of lactate dehydrogenase (LDH). The cells were treated with a dose of 12.5, 25, and 50 μM for 6 h [52]. Under the same conditions, RA symptoms in RAFLS were attenuated by repression of IL-1β, IL-6, NF-κB, ERK1/2, AP-1, and VEGF-A [38]. Curcumin, from rhizome of *Curcuma longa*, likewise demonstrated anti-RA effects via suppression of Bcl-2, caspase-3, caspase-9, ADP-ribose, and COX-2 of FLS when human FLS were treated for 24 h with a dose of 25, 50, 75, and 100 μM [53]. Curcumin oil-water nanoemulsions (CM-Ns) from the herb turmeric mitigated RA symptoms by downregulating NF-κB, TNF-α, and IL-1β in AIA rats which was treated with 50 mg/kg of CM-Ns for 24 h [54]. Emodin, extracted from *Rheum palmatum*, showed anti-RA effects on CIA rats that were given a dose of 10 mg/kg of the compound for 11 days, by inhibiting NF-κB, MMP, and M-CSF [55]. A dose of 5, 10, and 20 mg/kg on CIA rats for 21 days also demonstrated anti-RA effects via suppression of TNF-α, IL-6, and PGE2 [56]. On human synovial membrane which was administered emodin it the same effect by downregulating histone deacetylase (HDAC), HDAC1, VEGF, COX-2, COX-2, VEGF, hypoxia-inducible factor (HIF)-1a, MMP-1, MMP-13, NF-κB, and MAPK [57]. 

## 3. Discussion

Rheumatoid arthritis (RA) is an autoimmune disease that induces chronic joint inflammation, which causes cartilage and bone damage [1]. Synovial inflammation, swelling, autoantibody production, cartilage and bone destruction, and systemic features such as cardiovascular, pulmonary, and skeletal disorders are the main symptoms of this disabling autoimmune disease. Currently, non-steroidal anti-inflammatory drugs (NSAIDS), glucocorticoids, DMARDs, immunosuppressants, and biologic agents have been used to treat this autoimmune disease. DMARDs, especially, has been acknowledged as an effective early intervention for RA, their efficacy being validated by several randomized trials [58,59]. However, each DMARD showed different toxicity that causes side-effects such as diarrhea and rashes [59], and therefore various studies have been conducted to find a better solution for RA treatment. We saw the potential of finding the solution in natural products, especially polyphenols.

Flavonoids demonstrate anti-RA effects through diverse mechanisms [24]. α-Glucosyl-hesperidin showed results in an animal model study, but it lacked a specific discussion on the mechanism of the effect [27]. A study on cocoa polyphenol extract (CPE) discusses in depth the effect of the compound on several different inflammatory routes such as VEGF regulation, PI3K-Akt, and MAPK pathways [29]. Epigallocatechin-3-gallate (EGCG), from green tea is the most widely studied polyphenol related to RA. Lee et al. conducted an in-depth study on this compound using CIA rats and found specific elements that regulate and are regulated by Th17 cells and p-STAT3. This study further has observed gene-level events, which showed that control of *Nrf*2 gene may lead to anti-RA effects [32]. Yun et al., on the other hand, noted the mechanism of EGCG’s effect from a different perspective, comprehensively focusing on MMP production via the MAPK and AP-1 pathways [31]. Morinobu et al. focused their study on the role of nuclear factor of activated T cells c1 (NF-ATc1) in EGCG’s effect on osteoclasts [35]. On the other hand, a study by Leichsenring, et al. lacked a detailed discussion on the mechanism of EGCG [34]. A study of Oka, et al. on both EGCG and TFDG also gave an incomplete description of the mechanism of the compounds’ anti-RA effects [36]. A study on a flavonol-rich residual layer of hexane fraction (RVHxR) gave a poor examination of the role of elements in the MAPK pathway of angiogenesis [37]. A study on genistein by Zhang et al. gave a limited discussion of the possible mechanism of the compound’s effect on RA [40]. Umar et al. focused their studies on the effect of hesperidin on lipid peroxidation, which is another large category of RA pathogenic mechanisms. This research considered diverse lipid peroxidation factors, providing dense information about the effect of hesperidin in lipid peroxidation which causes RA [41]. Decendit et al. nicely designed a study on malvidin-3-*O*-b glucoside. The study included both animal model experiments, as well as animal and human cell experiments. The study also descriptively illustrated the malvidin-3-*O*-β-glucoside-related RA pathogenic pathway targeting macrophages [42]. Thymoquinone (TQ) was studied systematically in two studies. Vaillancourt, et al conducted an in-depth study on TQ, setting three stages of experiments, which included in vivo experiments on human RAFLS and a rat model and in vitro tests on an animal model. This study observed the effect of TQ on RA based on three different pathogenic pathways, which are lipid peroxidation, inflammation, and bone destruction. Interactions of elements that comprise each pathway are described in detail through an organized experiment process [48]. Umar et al, suggested a new point of view in studying the effects of polyphenols on RA pathogenesis. The study focuses on the role of apoptosis signal-regulating kinase 1 (ASK1) in the TNF-α signaling pathway and explains the role of its related factors in RA reduction [46]. In a study by Tekeoglu et al. three different experimental groups and a control group were used, but the results was unhelpful in explaining the molecular mechanism of the regulating effect of TQ [47]. 

Phenolic acids, plant metabolites that are widely spread throughout the plant kingdom, also possess anti-RA effects. A study on ferulic acid made profound observations on the effect of the compound in the RA pathogenic pathway, especially targeting the relation of RANKL, an osteogenic factor, and NF-κB signaling pathway [10]. Kwaket al. conducted an incomplete study on chlorogenic acid. Considering that RA’s pathological pathway contains various immunological factors, only using osteoclasts as the experiment cell line is limiting [13]. Neog et al. conducted a thoughtful study on the effect of *p*-coumaric acid (CA), also focusing on the system related to RANK and its interaction with T cell immune system factors [15]. Another study of CA, designed by Pragasam et al., was conducted under similar experimental conditions as used by Neoget al., but was imperfect in elucidating the molecular mechanism of CA’s anti-Ra effect [14].

Stilbenes are polyphenols that have two phenyl moieties connected by a two-carbon methylene bridge. Most of the studies on stilbenes that showed anti-RA effects were made on resveratrol. Three noticeable studies were made on resveratrol. Tsai et al. particularly noted resveratrol’s role in regulating COX-2 and PGE2 interaction. This study is unique because it focuses on the effect of particulate matter (PM) from air pollution on RA, and sees how resveratrol affects the inflammatory pathways of RA caused by PM [19]. Wahba et al. reported the effect of this compound from three perspectives. They observed immunological changes, inflammatory systemic changes, and oxidative stress changes. 

Choosing specific biomarkers for each part, this study specifically elucidated the role of resveratrol in each pathway [23]. A study by Xuzhu et al. observed three different levels of the object, which included the CIA animal model, DLN cells, and Th17 cells. Despite the effort to observe the result in diverse ways, this study failed to identify the specific mechanism of resveratrol’s effect on RA regulation at a molecular level [22]. 

In addition to flavonoids, phenolic acids, and stilbenes there are several other polyphenols that are hard to classify. Among them, curcumin (CM)-related molecules and emodin are the most actively studied polyphenols. Ramadan et al. systemically examined the anti-inflammatory and anti-oxidant effects of curcuminoids shrewdly considering diverse factors related to the pathways [51]. Kloesch et al. comprehensively tested the effect of CM on various inflammation pathway factors, but the duration of this study was too short [52]. A study by Zheng, et al. suggested a new way to increase the bioavailability of CA by forming CM-loaded Ns (CM-Ns). They also skillfully designed their experiments with three different experimental groups and one control group. However, a study on the molecular mechanism of CM-Ns’ anti-Ra effect was lacking [54]. Park et al. conducted an n in depth study on how emodin targets the apoptosis pathway, mainly focusing on Bax/Bcl-2 imbalance and activation of caspase-9 and caspase-3 [53]. A study of Ha, et al. on emodin thoroughly studied different aspects of the RA pathogenic pathway and tested the compound in vivo, which signified its role in inflammatory conditions [57]. Zhu et al. further conducted an animal model study on the effect of emodin on RA symptoms, but their explanation on the therapeutic mechanism in the pathogenic pathway was deficient [56].

**Figure 1:**
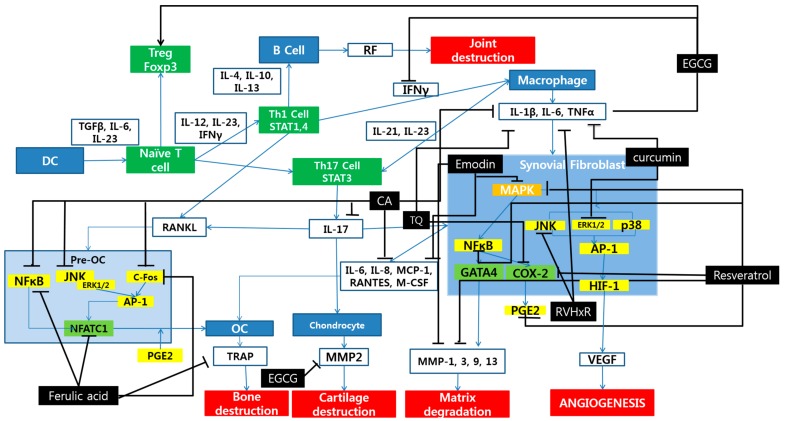
Schematic diagram of anti-inflammatory mechanisms of polyphenols. OC, osteoclasts; DC, dendritic cells; TQ, thymoquinone; CA, coumaric acid.

Polyphenol inhibit RA progress mainly by acting on three pathways: the inflammatory pathway, the oxidative pathway, and the apoptotic pathway. The inflammatory pathway regulated by polyphenols is mainly via the MAPK pathway and through regulation of NFATC1 gene in osteoblasts. The key molecules related to these processes are MAPK, IL-1β, IL-6, TNF-α, NF-κB, JNK, ERK1/2, AP-1 and COX-2 (Figure 1).

**Figure 2:**
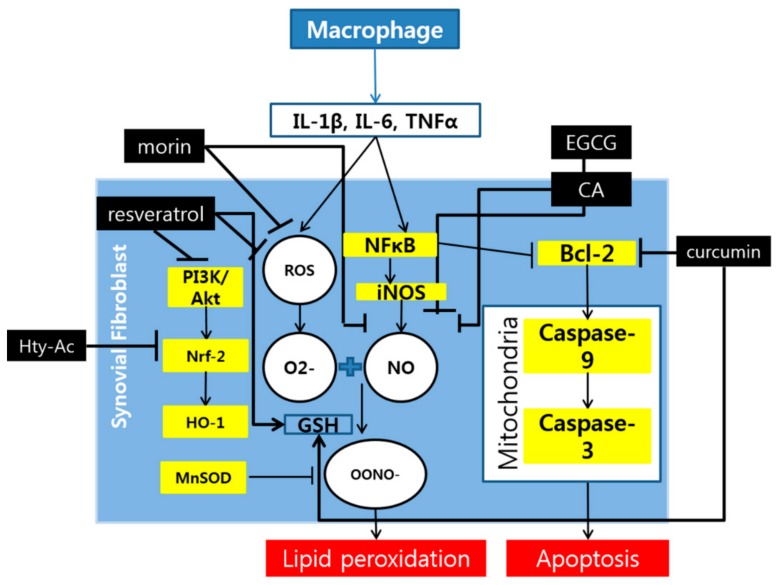
Schematic diagram of the anti-oxidative and apoptotic mechanisms of polyphenols.

Although they are not mentioned frequently among the studies, the oxidative and apoptotic pathways are also attributed a role in the reduction of RA symptoms by polyphenols (Figure 2). The key elements in the oxidative pathway which are controlled by polyphenols are mostly in the PI3-K/Akt pathway that produces HO-1 through transcription of the Nrf-2 gene. Other than this pathway, iNOS is frequently mentioned as the target of polyphenols. For the apoptotic pathway, only the pathway that involves Bcl-2 is indicated among many studies.

Studies on polyphenols’ anti-RA effects have mainly focused on their influence on inflammation pathways. There are some studies that concentrate on the anti-oxidative and apoptotic effect of polyphenols which result in a reduction of RA symptoms, but those are few in number. Further studies are needed in clarify the molecular studies mechanism of polyphenols’ anti-oxidative and apoptotic effects that regulate RA’s pathogenic pathways. 

In this review we have organized and summarized the role of each polyphenol compound in diverse pathogenic pathways of RA. This work will be significant in providing systematized information for developing natural-product-based RA therapeutic solutions.

## 4. Methods

Searches regarding the anti-RA effects of polyphenol were conducted on PubMed and Google Scholar in August of 2018. When searching for appropriate studies, we included “rheumatoid arthritis”, and “polyphenol” as keywords. Only articles written in English, published from 2006 to 2018 were selected for further review. We selected studies which met the following criteria: (i) studies based on in vitro or in vivo experiments that demonstrate the anti-RA effects of polyphenols; (ii) studies that show statistically significant analysis data (*p* < 0.05); (iii) studies that were not shown to have errors by subsequent studies; (iv) studies written in English. For classifying the type of polyphenol, we used the method of Soto et al. [60].

## 5. Conclusions

Polyphenols reduce rheumatoid arthritis symptoms by regulating an extensive collection of RA-related molecules, including MAPK, IL-1β, IL-6, TNF-α, NF-κB, JNK, ERK1/2, AP-1 and COX-2. Studies on polyphenols’ anti-RA effect were mainly focused on their influence on inflammation pathways. Further studies are needed for clarifying the molecular mechanism of polyphenol’s anti-oxidative and apoptotic effects that also regulate RA’s pathogenic pathways. Based on these preclinical data, clinical trials could be conducted.

## Figures and Tables

**Table 1:** Rheumatoid arthritis-inhibiting phenolic acids.

Compound	Source	Cell Line/Animal Model	Dose/Duration	Mechanism	Reference
Ferulic acid	Grains (rice, wheat and oats), vegetables, fruits, nuts	monocyte/macrophage cells/Rat	25, 50, 100 μM/24 h	↓ NFATc1, c-Fos, NF-κB, TRAP, MMP-9, Cathepsin	[10]
Natural polyphenol *N*-feruloylserotonin (*N*-f-5HT)	*Leuzea carthamoides*	AA	3 mg/kg/28 days	↓ CRP, LOX, TNF-α, iNOS, IL-1β	[11]
Gallotanins	*Euphorbia*	HMC-1/human	10 mg/mL/30 min	↓ TNF- α, IL-1β, IL-6, NF-κB	[12]
Kaempferol (3,5,7,4′-tetrahydroxy-flavone)	Gallic acid	RASFs/human	100 µM/ 2 days	↓ IL-1β, MMPs, COX, PGE2	[16]
Chlorogenic acid (CGA)	*Gardenia jasminoides*	osteoclast/ BMMs	10, 25, 50 μg/mM/4 days	↓ NF-κB, P38, Akt, ERK	[13]
*p*-Coumaric Acid (CA)	*Gnetm cleistostachyum*	AIA	100 mg/kg/8 days	↓TNF-α, CIC ↑ IgG	[14]
*p*-Coumaric Acid (CA)	*Gnetm cleistostachyum*	AIA	100 mg/kg/16 days	↓ TNF-α, IL-1β, IL-6, MCP-1, RANKL, TRAP, IL-1β, IL-6, IL-17, iNOS, COX-2, NF-κB-p65, p-NF-κB-p65, NFATc-1, c-Fos, JNK, p-JNK, ERK1/2 ↑OPG	[15]

AA, adjuvant arthritis; HMC-1, human mast cell line; RASFs, rheumatoid arthritis synovial fibroblasts; BMMs, bone marrow-derived macrophages; AIA, adjuvant induced *arthritis;* NFATc1, nuclear factor of activated T cells c1; NF-κB, nuclear factor κ light chain enhancer of activated B cells; TRAP, tartrate-resistant acid phosphatase; MMP-9, matrix metalloproteinases-9; CRP, c-reactive protein; LOX, 12/15-lipoxygenase; TNF-α, tumor necrosis factor-α; iNOS, inducible nitric oxide synthase; IL-1β, interleukin-1β; COX, cyclooxygenase; PGE2, prostaglandin E2; CIC, circulating immune complexes; IgG, immunoglobulin G; MCP-1, monocyte chemoattractant protein-1; RANKL, receptor activator of nuclear factor kappa-B ligand; TRAP, tartrate-resistant acid phosphatase; JNK, c-Jun N-terminal kinases; OPG, osteoprotegerin; ↑—up-regulation; ↓—down-regulation.

**Table 2:** Rheumatoid arthritis-inhibiting stilbenes

Compound	Source	Cell Line/Animal Model	Dose/Duration	Mechanism	Reference
Resveratrol	Red grapes	FLSs/AA	5, 15, 45 mg/kg/12 days	↑ MtROS ↓ Beclin1, LC3A/B, MnSOD	[18]
Resveratrol	Red grapes	FLSs/Human	50 μg/24 h	↓ COX-2, PGE2, NADPH oxidase, ROS, Akt, p38, MAPK, ERK1/2, NF-κB	[19]
Resveratrol	Red grapes	FLSs/Human	6.25, 12.5, 25, 50 µM/1 h	↓ IL-1β, MMP-3, P-Akt, PI3K-Akt	[20]
Resveratrol	Red grapes	Human * randomized controlled clinical trial	1000 mg/day/3 month	↓ RF, MMP-3, TNF-α, IL-6,	[21]
Resveratrol	Red grapes	1) CIA 2) DLN cell/CIA 3) Th17 cell/CIA	1) 20 mg/kg/10days 2) 40 µM/72 h 3) 30 µM, 50 µM/3 days	1) ↓ IgG1, IgG2a 2) ↓ IL-17, IFN-γ 3) ↓ TH-17, IL-17	[22]
Resveratrol	Red grapes	CFA induced rat	10 mg/kg/day/7 days	RF, MMP-3, COMP, IgG, ANA, TNF-a, MPO, MDA ↑ IL-10, GSH	[23]

FLSs, fibroblast-like synoviocytes; CIA, collagen-induced arthritis; DLN, draining lymph node; CFA, cetylated fatty acids; MtROS, mitochondrial ROS; LC3, microtubule-associated protein 1a/1b-light chain 3; MnSOD, manganese-dependent superoxide dismutase; COX, cyclooxygenase; PGE2, prostaglandin E2; NADPH, nicotinamide adenine dinucleotide phosphate; ROS, reactive oxygen species; MAPK, mitogen-activated protein kinase; NF-κB, nuclear factor κ light chain enhancer of activated B cells; IL-1β, interleukin-1β; MMP-3, matrix metalloproteinases-3; PI3K, phosphoinositide 3-kinases; RF, rheumatoid factor; TNF-α, tumor necrosis factor-α; IgG, immunoglobulin G; IFN, interferon; COMP, cartilage oligomeric matrix protein; ANA, antinuclear antibodies; MPO, myeloperoxidase; MDA, malondialdehyde; GSH, glutathione; ↑—up-regulation; ↓—down-regulation.

**Table 3:** Rheumatoid arthritis-inhibiting flavonoids

Compound	Source	Cell Line/Animal Model	Dose/Duration	Mechanism	Reference
A-glucosylhesperidin	Citrus fruit	CIA rat	3 mg/0.3 mL/3 times a week, 31 days	↓ TNFα	[27]
Anthocyanin	Cherries	AIA rat (Male Sprague Dawley)	10, 20, 40 mg/kg/14 days	↓TNFα, PGE2, MDA ↑ SOD	[28]
Cocoa polyphenol (epicatechin, catechins, flavonol glycosides and procyanidin)	Cocoa	JB6 P+ mouse epidermal cells	0, 10, 20 μM /mL/1 h	↓ VEGF, NF-kB, AP-1 ↓ p-Akt, p-p70S6K, p- ERK, p- p90RSK, p- MKK4, p-JNK, p- PI3K	[29]
Epigallocatechin-3-gallate (EGCG)	Green tea (*Camellia sinensis*)	RASFs	10, 20, 30, 40, 50 μM/12 h	↓ENA-78, RANTES, GRO-alpha, MMP-2	[30]
Epigallocatechin-3-gallate (EGCG)	Green tea	CIA rat (DBA/1J)	20, 30, 40, 50 mg/kg/3 weeks	↓ IgG2a, IL-1β, IL-6, TNFα, TRAP, IL-17, VEGF, nitrotyrosine, iNOS, p-STAT3, c-Fos, NFATc1, CTSK, MMP9, p-STAT3 727, IL-17, CCL6, AHR, IL-21, p-STAT3 705, p-ERK, RANK, CTR ↑ IL-10, TGF- β, SOCS3, Foxp3	[32]
Epigallocatechin gallate	Green tea	PIA rats (Dark Agouti)	10 mg/kg/5 days	↓ MPO	[34]
Epigallocatechin-3-gallate (EGCG)	Green tea (*Camellia sinensis*)	1) Human osteoclasts of peripheral blood monocytes 2) DBA/1 mice	20 μM, 50 μM/15 days	↓ CTR, carbonic anhydrase II, cathepsin K, alpha-v integrin, β-3 integrin, NF-ATc1	[35]
Epigallocatechin-3-gallate (EGCG)	Green tea	CIA rat (DBA/1J)	10 mg/kg/3 weeks	↓ IL-6, TNFα, IFN-γ ↑anti-CII specific IgG1 antibodies	[33]
Epigallocatechin-3-gallate (EGCG)	*Camellia sinensis*	Osteoclast precursors cells mature osteoclasts	10, 100 μM/7 days	↓ Multinucleated osteoclast formation, MMP-9, MMP-2	[36]
Epigallocatechin 3-gallate (EGCG)	Green tea (*Camellia sinensis*)	RASFs	125, 250, 500 nM/24 h	↓ MAPK, MMP-1, MMP-3, p-ERK1/2, p-JNK, p-p38, AP-1	[31]
Fisetin	*Rhus verniciflua* Stokes	RA FLs	0.1, 1, 10 μg/mL/72 h	↓ TNFα, IL-6, IL-8, MCP-1, VEGF	[37]
Flavonol-rich residual layer of hexane fraction (RVHxR)	*Rhus verniciflua* Stokes	RA FLs	0.1, 1, 10 μg/mL/72 h	↓ TNFα, IL-6, IL-8, MCP-1, VEGF ↓ p-ERK, p-JNK, ↑ p- p38-MAPK	[37]
Gallic acid	*Cinnamomum zeylanicum* Bark	1) AIA rat 2) Concanavalin (Con-A) stimulated lymphocyte	1) 200 mg/kg/12 days, 21 days 2) 40 μg/100 μL/72 h	1) ↓ TNF-α 2) ↓ IL-2, IL-4, IFN-γ	[38]
Genistein		CIA rats	1 mL/kg/42 days	↓ IFN-γ, Th1/Th2, T-bet ↑ GATA-3, IL-4	[39]
Genistein	Soybean	RA FLS	10 μg/mL/24 h	↓ MMP-9	[40]
Hesperidin		CIA rat (Wistar rat)	160 mg/kg / 22 days	↓ ELA, TBARS, nitrite ↑ GSH, SOD, catalase	[41]
Malvidin-3-O-β-glucoside	Red grape skinExtract powder	1) peripheral blood monocyte-derived macrophages 2) peritoneal macrophages 3) AIA rat	1) 1, 10, 100 μM/24 h 2) 1, 10, 100 μM/24 h 3) 25 mg/kg/10 days	1) ↓ TNF-α, IL1, MIP1a, IL-8, IL-6, NO, NOx 2) ↓ IL-1β, TNF-α, IL-8	[42]
Mangiferin	Thymelaeaceae family (e.g., *Phaleria cumingii*)	CIA rat (DBA/1)	100 and 400 mg/kg/14 days and 27 days	↓ NF-κB, ERK1/2,IL-1β, IL-6, TNF-α, RANKL	[43]
Morin (ML-morin)	Fruits, vegetables, tea	Spleen and synovial macrophages	10 mg/kg/3 days	↓ ROS, NO, iNOS, NF-κB-p65, TNF-α, IL-1 β, IL-6, MCP-1, VEGF, RANKL, STAT-3	[44]
Naringin	Grape, citrus fruit	AIA rat (Female Sprague-Dawley)	1) 20 mg/kg/28 days 2) 40 mg/kg/28 days	↓ TNFα, IL-1β, IL-6, Bcl-2 ↑ Bax	[45]
Theaflavin-3,3′-digallate (TFDG)	*Camellia sinensis*	osteoclast precursors cells mature osteoclasts	10, 100 μM/7 days	↓ Multinucleated osteoclast formation, MMP-9, MMP-2	[36]
Thymoquinone	*Nigella sativa*	RA synovium	1, 2, 3, 4, 5 μM/2 h	↓ IL-6, IL-8, ICAM-1, VCAM-1, Cad-11, p38, JNK	[46]
Thymoquinone	*Nigella sativa*	CIA rat (Sprague-Dawley Wistar rat)	2.5 mg/kg/5 days 5 mg/kg/5 days	↓ IL-1β	[47]
Thymoquinone	*Nigella sativa*	1) RA FLS 2) AIA rat	1) 0, 1, 2, 5, 10 μM/1 h 2) 5 mg/kg/1 day	1) ↓LPS-induced IL-1β, TNFα, MMP-13, COX-2, prostaglandin, HNE, p-p38-MKK, p-ERK, p-NF-κB-p65 2) ↓ HNE, IL-1β, TNFα	[48]

RA FLS, RA fibroblast-like synoviocytes; MKK4, mitogen-activated protein kinase kinase 4; ENA-78, Epithelial neutrophil- activating protein 78; RANTES, regulated on activation, normal t cell expressed and secreted, GRO, growth-regulated oncogene; VEGF, vascular endothelial growth factor; AHR, aryl hydrocarbon receptor; ICAM, intercellular adhesion molecules; VCAM, vascular cell adhesion protein; LPS, lipopolysaccharides; HNE, H_2_O_2_-induced 4-hydroxynonenal; ↑—up-regulation; ↓—down-regulation.

**Table 4:** Rheumatoid arthritis-inhibiting other polyphenols

Compound	Source	Cell Line/Animal Model	Dose/Duration	Mechanism	Reference
EVOO-polyphenol extract (PE)	EVOO	CIA in DBA-1/J	100, 200 mg/kg/13 days	↓ TNF-α, IL-1β, IL-6, PEG2, p38, JNK, p65, lκB- α	[49]
Hydroxytyrosol acetate (Hty-Ac)	EVOO	CIA in DBA-1/J	0.05%/42 days	↓ IgG1, IgG2a, COMP, MMP-3, TNF-Q, IFN-S, IL-1R, IL-6, IL-17A, Nrf2, HO-1	[50]
Curcuminoid	Turmeric rhizome Ginger rhizome	AIA	200 mg/kg/28 days	↑ TNF-α, IL-1β, IL-6, IL-4, IL-10, SOD, CAT, GSH ↓ LPO, ALAT, ALP	[51]
Curcumin	Turmeric rhizome	1) MH7A 2) RA-FLS	12.5, 25, 50 μM/6 h	1) ↓ IL-1β, IL-6, NF-κB, ERK1/2, AP-1 ↑LDH 2) ↓ IL-1β, IL-6, NF-κB, ERK1/2, AP-1, VEGF-A	[52]
Curcumin oil-water nanoemulsions (CM-Ns)	Herb turmeric	AIA	50 mg/kg/14 days	↓ NF-κB, TNF-α, IL-1β	[54]
Curcumin	Rhizome of *Curcuma longa*	FLS/Patient	0, 25, 50, 75, 100 μM/24 h	↓ Bcl-2, COX-2↑caspase-3, caspase-9	[53]
Emodin	*Rheum palmatum*	CIA DBA/1 J	10 mg/kg/11 days	↓ NF-κB, MMP, M-CSF	[55]
Emodin	*Rheum palmatum*	CIA	5, 10, 20 mg/kg/21 days	↓ TNF-α, IL-6, PGE2	[56]
Emodin	*Rheum palmatum*	Synovial membrane/Humans	0.1, 1, 10 μM/24 h	↓ HDAC, HDAC1, VEGF, COX-2, HIF-1a, MMP-1, MMP-13, NF-κB, MAPK	[57]

MH7A, rheumatoid synovial cell; Nrf2, nuclear factor (erythroid-derived 2)-like 2; HO-1, heme oxygenase 1; LPO, lipid peroxidation; ALAT, alanine transaminase; ALP, alkaline phosphatase; LDH, lactate dehydrogenase; M-CSF, macrophage colony-stimulating factor; HDAC, histone deacetylase; HIF, hypoxia-inducible factor; ↑—up-regulation; ↓—down-regulation.
